# Does the addition of concurrent visual feedback increase adherence to a home exercise program in people with stroke: a single-case series?

**DOI:** 10.1186/s13104-020-05202-2

**Published:** 2020-07-29

**Authors:** Tamina Levy, Maria Crotty, Kate Laver, Natasha Lannin, Maggie Killington

**Affiliations:** 1 Flinders Health and Medical Research Institute, Flinders University, Sturt Road, Bedford Park, 5042 Australia; 2 Flinders Medical Centre, Rehabilitation and Palliative Services, Bedford Drive, Bedford Park, SA 5042 Australia; 3 Department of Neuroscience, Central Clinical School, Monash University, Melbourne, Australia; 4 Occupational Therapy Department, Alfred Health, Melbourne, Australia; 5 SA Brain Injury Rehabilitation Services, Royal Adelaide Hospital, Adelaide, SA Australia; 6 Physiotherapy Department, Flinders Medical Centre -RAP Division, Bedford Drive, Bedford Park, SA 5041 Australia

**Keywords:** Stroke, Technology, Feedback, Exercise

## Abstract

**Objective:**

Evidence is accumulating for the potential benefits of technology use in stroke rehabilitation. However, few studies have examined ways in which technology can be used to increase adherence to programs after discharge from rehabilitation. The aim of this study was to determine if the addition of concurrent visual feedback, via a tablet computer, increased adherence to an exercise program following stroke. Ten participants were provided with a self-administered exercise program and were asked to perform 60 min of the exercises daily. After a baseline phase (1 week), participants were given a tablet computer (2 weeks) and were asked to video record each exercise session. The tablet computer was removed during the fourth week of the program.

**Results:**

Exercise duration, measured via wrist-worn accelerometry, was investigated over the 4 weeks using the two-standard deviation (2 SD) band method. A statistically significant effect was observed in four out of ten cases, demonstrated by two successive data points occurring outside the 2 SD band during the intervention phase, suggesting that adherence was increased in response to the tablet computer use. This preliminary study indicates that the use of visual feedback, via a tablet computer, may increase adherence to an exercise program in people with stroke.

*Trial registration* ACTRN: ACTRN12620000252910 (26 February 2020, Retrospectively registered)

## Introduction

The use of tablet devices to increase engagement in rehabilitation is increasing as services have greater access to technology [1]. Tablet computers, such as iPads^®^, are portable and inexpensive and many individuals own these devices [2].

Whilst there is an increasing number of applications installed on tablet computers to increase participation in therapy, there is a lack of research around use of tablet computers as a means of video recording participation in therapy and providing real-time feedback. In a review of tablet use in stroke rehabilitation, Ameer and Ali described benefits in using a device with a camera within and outside of therapy; including allowing the therapist to record sessions and provide real-time feedback [3]. This finding is consistent with an exploratory study using video feedback of functional tasks after stroke, which found that participants who were provided with visual feedback during a task expressed greater satisfaction [4]. There is a lack of studies investigating the role real-time feedback, via tablet use, may play in promoting exercise adherence following stroke, and this should be further explored.

Technology devices can also be utilised to monitor adherence. Adherence has been described as ‘the extent to which a person’s behaviour—taking medication, following a diet, and/or executing lifestyle changes, corresponds with agreed recommendations from a healthcare provider’ [5]. Researchers and clinicians require accurate methods to measure adherence. However, measuring adherence to exercise is challenging, and consensus regarding the gold standard is lacking [6, 7]. Accelerometers are wearable sensors, designed to measure movement in activity counts [8]. The advantage of accelerometers is that an objective measure can be gained. There is evidence that accelerometers produce reliable and valid metrics of upper limb use [9, 10] and the feasibility of using accelerometers to monitor exercise adherence should be explored.

The primary aim of this study was to determine if the addition of concurrent visual feedback, via a tablet computer, would increase adherence to an upper limb home exercise program in people with stroke.

A secondary aim was to assess the feasibility of use of accelerometers as a method of monitoring upper limb activity during a home exercise program in people with stroke.

## Main text

### Methods

Ethical approval for this study was granted by The Southern Adelaide Clinical Human Research Ethics Committee (SAC HREC EC00188). Ten participants were recruited from the Flinders Medical Centre. Participants provided written informed consent. Inclusion criteria were diagnosis of stroke (> 3 months), completion of rehabilitation, and being able to pick up a small block with the stroke-affected hand. Patients were excluded if they were less than 18 years old, had a Mini-Mental State Examination score (MMSE) of less than 24 out of 30 [11], were non-English speaking, or had visual deficits preventing technology use. Baseline outcome measures were collected to provide an understanding of demographics. Measures were Fugl-Meyer Assessment (FMA) [12], Modified Rankine Score (0–6) [13], Motor Activity Log-14 (MAL-14) [14], Self-Efficacy for Exercise Scale [15], Box and Block Test (BBT) [16], line bisection test [17] and the Technology Use Questionnaire (developed by authors, see Additional file 1: Appendix S1). This study adheres to the CONSORT guidelines for clinical trials (Additional file 2).

A single-case series design was employed [18] with an A–B–A design. A single-case series design allows for detailed testing of the efficacy of an intervention on a chosen outcome. In single-case experiments, sequential measurements are recorded for each participant. After an initial baseline period, an intervention is applied and the effect of this intervention relative to the baseline is investigated. Following the intervention phase, there is a follow-up phase with withdrawal of the intervention.

Participants were visited at home by the researcher. Following completion of baseline measures, the participants were instructed in the Graded Repetitive Arm Supplementary Program (GRASP) [19] and were asked to practice their program for a total of 60 min, daily for 4 weeks. The GRASP program, a self-directed arm and hand exercise program which has been shown to improve function after stroke, prescribes 1 h of daily exercises and a manual of exercises is provided [19]. Participants were provided with a recording sheet to record the time spent exercising during each session.

#### Intervention (tablet computer use)

During the intervention phase (weeks 2 and 3), participants were provided with a tablet computer (Apple model A1474) and were asked to video record each session, using the MoviePro App, a videorecording app that was downloaded onto the tablet computer for a small fee paid by the researchers. The tablet computer was set up in front of the participant so that they were able to see themselves exercising and they were instructed to look at the screen as they exercised. Participants also recorded the start and finish times of each session on a recording sheet.

#### Measurements

Participants were provided with two wrist-worn accelerometers (Actigraph) and were instructed to don these prior to their exercise session and to remove them at the completion of each session. The Actigraph, a lightweight accelerometer that resembles a wristwatch, measures movement of the upper limb through acceleration.

The outcome measure used to evaluate adherence in this study was active time when wearing the accelerometer. At the completion of the study the researcher downloaded recordings from the Actigraph devices. Data was analysed using the Actilife Software, and active time was calculated for each session.

To evaluate the feasibility of accelerometry use to measure adherence, The System Usability Scale (SUS), a 1 to 5 Likert scale that measures participants' experience using technology was completed [20]. Additionally, the researcher kept a logbook, recording any issues that arose.

#### Data analysis

Changes in amount of activity recorded on the accelerometers from the baseline phase to the “tablet intervention” phase provided an indication of whether adherence to exercise increased in response to the tablet. Additionally, changes in accelerometer recorded activity following tablet removal in the follow-up phase provided extra information to inform interpretation of the data.

Following standards related to single-case series research where the participant acts as their own control, 5 measures were analysed in the baseline phase and the mean and standard deviation of measures were calculated to account for the level (mean score) and trend (slope) of the 5 measures prior to introduction of the tablet [21]. Active wear time data were then analysed using the two standard deviation (2 SD) band method which has been recommended for analysis of single-case series designs [18, 22, 23]. If two or more successive data points within the intervention phase fell outside the 2 SD band (i.e. outside the 95% confidence limits), changes from baseline to intervention were considered statistically significant. Rigour of the methodology was enhanced by replication of the design on 10 different occasions with 10 participants. This method of evaluation enables performance variability to be factored into the analysis [24].

### Results

Table 1 presents the baseline demographics of participants.

**Table 1:** Subject demographics at baseline

Subject	Age	MMSE	FMA	MAL14	Self-efficacy	MRS	BBT (affected)	BBT (un affected)	Time since stroke (months)	Time since rehabilitation (months)
1	64	26	63	7.2	6.4	2	16	29	58	20
2	52	29	62	5.6	10.0	2	65	81	6	4
3	63	28	62	5.6	8.5	3	32	59	13	6
4	65	27	32	5.0	5.9	3	0	57	24	21
5	65	30	62	4.5	7.4	3	19	63	14	9
6	70	29	33	4.7	10.0	2	2	68	7	3
7	21	30	55	4.7	6.1	2	20	32	110	57
8	62	30	64	7.1	7.5	2	45	64	24	.5
9	56	30	65	7.9	9.7	2	48	54	3	1
10	63	30	35	5.1	7.3	3	36	60	5	4

*MMSE* Mini-Mental State Examination (0–30), *FMA* Fugl-Meyer Assessment (0–66), *MAL14* Motor Activity Log-14 (0–10), *Self*-*Efficacy* Self-efficacy for Exercise Scale (0–10), *MRS* Modified Rankine Score (0–6), *BBT* Box and Block Test

#### AIM 1: to determine if the addition of concurrent visual feedback, via a tablet computer, will increase adherence to an upper limb home exercise program in people with stroke

**Fig. 1:**
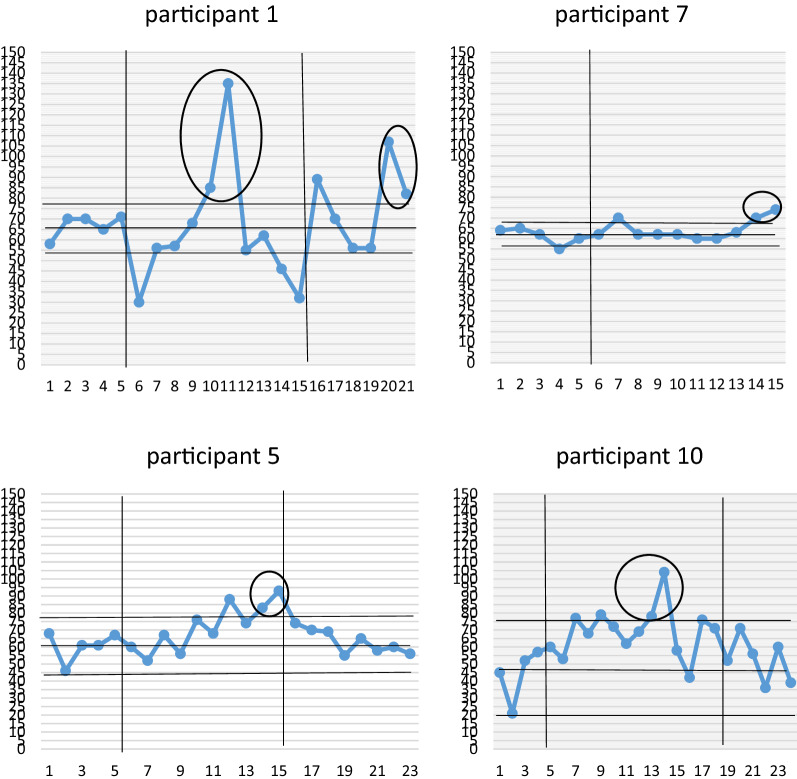
Data points through baseline, intervention and follow-up for Participants 1, 5, 7 and 10 (significant results circled with 2 consecutive data points outside 2 SD range). X axis represents days of exercise. Y axis represents exercise duration (minutes)

Overall, a significant effect was observed in 4 out of the 10 cases (participants 1, 5, 7, 10), as demonstrated by 2 successive data points occurring outside the 2 SD band during the intervention phase, meaning that these participants performed a significantly greater amount of exercise when they were using the tablet computer. These results are represented in Fig. 1.

**Fig. 2:**
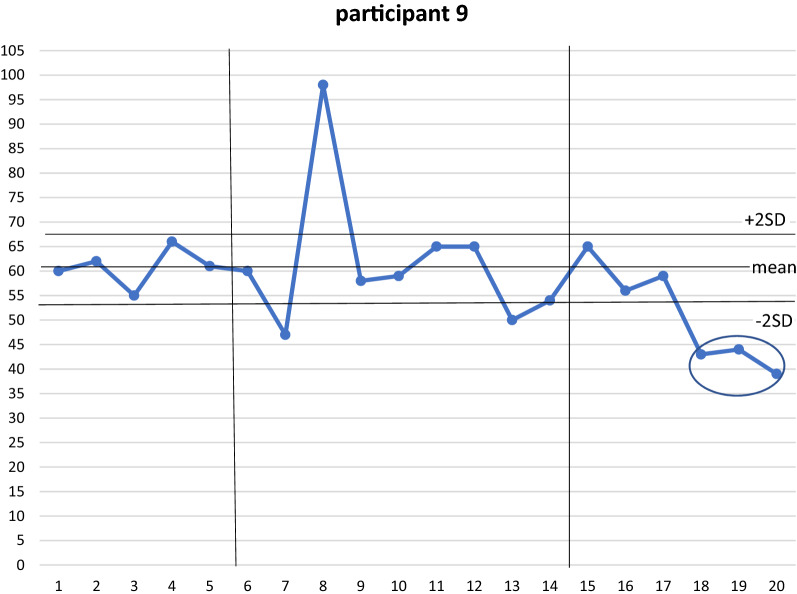
Data points through baseline, intervention and follow-up for Participant 9 (significant result circled with 2 consecutive data points outside 2 SD range). X axis represents days of exercise. Y axis represents exercise duration (minutes)

Furthermore, one participant (participant 9) showed a statistically significant reduction in performance at follow-up when the tablet computer was removed (Fig. 2).

Nine participants reported that they enjoyed the tablet computer and found it beneficial for giving feedback and improving engagement. Participant 1 reported that he did not like the experience as he felt like he was being watched, however he still self-reported a perception that the tablet use improved his adherence.

When time since stroke was investigated, the two participants with the longest time since their strokes (participant 1 = 58 months; participant 7 = 110 months), both showed a statistically significant change. Furthermore, when level of motor ability was explored, 4 of the 6 non responders to the intervention (participants 2, 3, 8 and 9) had recorded a relatively high Box and Block Test score.

#### AIM 2: to assess the feasibility of use of upper limb accelerometry as a method of monitoring upper limb activity

The mean score for the System Usability Scale was 96.5 out of 100, indicating a high level of usability. There were several problems in terms of data collection. Issues that arose included missing data (participants 4, 9, 10); despite reportedly charging the devices, data were missing during the last three days of exercise in Participants 4 and 9. Two participants forgot to put devices on and/or off (participants 2 and 5), on one occasion for each participant. A further two participants forgot to charge the accelerometers (participants 1 and 7), and participant 7 had no recorded data after day 15. Two participants were unable to put the device on the non-affected wrist without assistance (participants 4 and 6).

No issues arose with accelerometry utility or data collection in participants 3 and 8.

### Discussion

This study demonstrated that using a tablet computer as a tool to promote adherence (via real-time feedback) to an upper limb home exercise program can be useful for some people with stroke. Clinicians should assess individual patient factors such as level of motivation, familiarity with technology, and level of motor impairment when considering this method of technology use. A significant improvement in the amount of exercise performed was observed in four of the 10 participants. Additionally, a further participant showed a statistically significant reduction in performance at follow-up when the tablet computer was removed.

Most participants reported positive feelings towards the approach. This is consistent with findings of Gilmore and Spaulding who reported greater satisfaction in participants who received video feedback during a functional task [4]. Furthermore, in a randomised controlled trial investigating adherence to exercise in people with stroke, Emmerson et al. [25] compared paper-based home exercises to home exercises filmed on a tablet. The authors stated that a potential benefit could be the feedback aspect of the video use and suggested that this may be evaluated further.

There were no technical issues reported with tablet computer use and all participants managed to operate the devices without any assistance or with minimal carer assistance. This aligns with qualitative data that reported tablet computers are easy to use, acceptable and engaging [26].

A ceiling effect was observed in participants who were highly motivated; meaning there was less ‘room for improvement’, and hence no statistically significant effect occurred during the intervention. Testing the effect of the intervention on the adherence of a less motivated group of participants would be valuable. Four of the participants who did not show a significant change with the intervention were those who had recorded higher scores on the Box and Block Test. It may be that the visual feedback provided by the tablet computer is more sought out, utilised and beneficial when a patient has less motor control. The two participants in this study who presented at the longest time since stroke demonstrated a significant change with the intervention. It may be that this technology is most effective when patients are in the chronic phase of recovery, but this needs to be considered with caution.

The two participants in this study who had greater motor impairment required assistance to put the accelerometers onto their non-affected wrists. Some issues with utility arose, including participants forgetting to remove and charge the accelerometers. Interventions to promote reliability of accelerometry use should be considered and may include scheduling applications and phone text reminders. This small study has demonstrated that there are issues that may reduce utility of home-based accelerometry use in people with stroke. The main advantage of using the accelerometers were that they provided accurate data on exercise time.

## Limitations

The study sample was small and could be considered already motivated, having consented to a 4-week exercise program. A qualitative component would have enabled a greater exploration of participants’ experiences.

## Supplementary information

**Additional file 1: Appendix S1. **Technology Use Questionairre, developed by the authors.

**Additional file 2. **Consort checklist.

## Data Availability

The data that support the findings of this study are available on request from the corresponding author, TL. The data are not publicly available due to ethical restrictions (data may compromise the privacy of research participants).

## Abbreviations

GRASP: Graded Repetitive Arm Supplementary Program
SUS: System Usability Scale
2 SD: two standard deviation band method
