# Supplementary material for: Does the addition of concurrent visual feedback increase adherence to a home exercise program in people with stroke: a single-case series?
Source: BMC Res Notes. 2020 Jul 29;13:361. doi: 10.1186/s13104-020-05202-2 (PMC7391818; doi:10.1186/s13104-020-05202-2)
Supplement: Supplementary file 1 — Additional file 1: Appendix S1. Technology Use Questionairre, developed by the authors. [file 13104_2020_5202_MOESM1_ESM.docx]

**TECHNOLOGY QUESTIONNAIRRE**

**The following questionnairre is asking about how often you use everyday technology.**

**Please tick in the relevant column.**

|  | More than once a day | More than once a week | More than once a month | Rarely, or more than once a year | Never |
| --- | --- | --- | --- | --- | --- |
| Search for information on the internet |  |  |  |  |  |
| Use the TV remote control |  |  |  |  |  |
| Withdraw money from the automatic teller machine |  |  |  |  |  |
| Deal with recorded telephone me  nus |  |  |  |  |  |
| Tape a TV program using a recording device |  |  |  |  |  |
| Send and receive emails |  |  |  |  |  |
| Use a mobile phone |  |  |  |  |  |
| Operate a telephone answering service such as an answering machine or voicemail |  |  |  |  |  |
| Use a microwave oven |  |  |  |  |  |
